# Relationship between rheumatoid arthritis and cardiovascular comorbidity, causation or co-occurrence: A Mendelian randomization study

**DOI:** 10.3389/fcvm.2023.1099861

**Published:** 2023-03-17

**Authors:** Min Wang, Ce Chao, Kun Mei, Dongmei Di, Yongxiang Qian, Bin Wang, Xiaoying Zhang

**Affiliations:** Department of Cardiothoracic Surgery, The Third Affiliated Hospital of Soochow University, Changzhou, China

**Keywords:** rheumatoid arthritis, ischemic heart disease, myocardial infarction, atrial fibrillation, arrhythmia, Mendelian randomization analysis, genome-wide association study

## Abstract

**Background:**

In recent years, the incidence rates of rheumatoid arthritis (RA) and heart disease (HD) have noticeably increased worldwide. Previous studies have found that patients with RA are more likely to develop HD, while the cause and effect have still remained elusive. In this study, Mendelian randomization (MR) analysis was used to indicate whether there was a potential association between RA and HD.

**Methods:**

Data of RA, ischemic heart disease (IHD), myocardial infarction (MI), atrial fibrillation (AF), and arrhythmia were based on the genome-wide association study (GWAS) dataset. No disease group was intersected. Inverse-variance weighted (IVW) method was used to calculate MR estimates, and sensitivity analysis was performed.

**Results:**

The primary MR analysis showed that genetic susceptibility to RA was significantly associated with the risk of IHD and MI, rather than with AF and arrhythmia. Besides, there was no heterogeneity and horizontal pleiotropy between the primary and replicated analyses. There was a significant correlation between RA and the risk of IHD (odds ratio (OR), 1.0006; 95% confidence interval (CI), 1.000244–1.00104; *P* = 0.001552), meanwhile, there was a significant correlation between RA and the risk of MI (OR, 1.0458; 95% CI, 1.07061–1.05379; *P* = 0.001636). The results were similar to those of sensitivity analysis, and the sensitivity analysis also verified the conclusion. Furthermore, sensitivity and reverse MR analyses suggested that no heterogeneity, horizontal pleiotropy or reverse causality was found between RA and cardiovascular comorbidity.

**Conclusion:**

RA was noted to be causally associated with IHD and MI, rather than with AF and arrhythmia. This MR study might provide a new genetic basis for the causal relationship between RA and the risk of CVD. The findings suggested that the control of RA activity might reduce the risk of cardiovascular disease.

## Introduction

Ischemic heart disease (IHD) is the leading cause of death globally. In 2019, there were 197.2 million IHD cases and 9.1 million IHD-related deaths worldwide (1, 2). However, the prevalence and mortality rates of IHD have decreased over the past decades (1, 3). IHD treatment has shown to play a vital role in reducing the mortality rate of cardiovascular disease (CVD) and also contributed to the achievement of the Sustainable Development Goals (4). Smoking is the main cause of IHD morbidity, and metabolic risk factors may noticeably affect IHD morbidity, including hypertension, increased fasting plasma glucose (FPG) level, increased low-density lipoprotein-cholesterol (LDL-C) level, obesity, lack of exercise, and poor eating habits (1, 5). Consequently, metabolic risk factors will increase the prevalence rate of IHD in the future. IHD-associated risk factors may also cause rheumatoid arthritis (RA), and rheumatoid factors have been proved to be independent risk factors for IHD (6, 7). In a previous study, RA patients were compared to a normal population, and it was found that 48% of RA patients were more likely to develop CVD and 50% were likely to die from it (8–10). Not only RA and IHD have exhibited similar causes, but also IHD was reported as the leading cause of cardiovascular complications in RA patients (11). The use of biological disease-modifying antirheumatic drugs might be associated with the reduced risk of cardiovascular mortality in patients with RA. However, different drugs had different effects on the cardiovascular risk (12). The exact causal relationship between RA and CVD has still remained elusive.

Mendelian randomization (MR) can estimate the causal relationship without bias, and it has been used to explore the risk factors for CVD, such as arachidonic acid (13), linoleic acid (14), glucagon (15), body mass index (16), etc. Moreover, MR analysis has revealed the causality between atrial fibrillation (AF) (17) and thyroid function (18) with IHD. In previous studies, the relationship between RA and IHD has been tentatively explored. A study showed that RA patients were at the higher risk of heart failure (HF) and IHD than the general population and a population with osteoarthritis (19, 20). Due to the improvement of RA management, a decrease in cardiovascular mortality rates was recorded after 2004 (21).

However, RA patients could be associated with an increased risk of HF and it was irrelevant to the risk of IHD. This could be resulted from the rapidly increased risk of non-ischemic HF after RA onset (22). In the present study, the causal relationship between RA and IHD was analyzed using inverse-variance weighted (IVW) and MR-Egger methods.

## Methods

### Data sources and study design

A two-sample MR study was performed using a classical MR analysis scheme. Genome-wide association study (GWAS)-based data included summary-level data for RA that covered 14,361 cases and 43,923 controls, as well as single-nucleotide polymorphisms (SNPs) for AF as the outcome dataset that included 970,216 controls and 60,620 cases.

GWAS-based data for myocardial infarction (MI) as an outcome dataset were obtained from FinnGen (https://www.finngen.fi/en), which included 187,840 controls and 12,801 cases. Additional data were derived from a GWAS containing summary-level data for arrhythmia that covered 2,545 cases and 460,388 controls and summary-level data for IHD that included 5,861 cases and 457,149 controls. Demographic characteristics of cases are shown in Table 1.

**Table 1:** Assessment of instrumental variables and data sources.

Traits	Sample size (cases/controls)	Data sources	Ancestry	*R*^2^(%) for RA/AD (Total)	F for RA/AD (Total)
Exposure to RA	14,361/43,923	PMID: 33310728	European		
**Outcomes**
Arrhythmia	2,545/460,388	ukb-b-3703	European	0.0466	49
AF	60,620/970,216	PMID: 30061737	European	0.0611	44
MI	12,801/187,840	finn-b-I9_MI	European	0.0588	44
IHD	5,861/457,149	ukb-b-8184	European	0.0484	48

SD = SE × N^1/2^, where SE, the standard error of the estimated effect, *N*, the sample size of the GWAS. *R*^2^ = 2 × (1-EAF) × EAF × (b/SD)^2^, where b, the estimated effect on adipokines, EAF, the effect allele frequency. F = *R*^2^(N-K-1)/[K(1-*R*^2^)].

A two-sample MR analysis was carried out to assess the causal relationship between the risk of CVD and genetic susceptibility to RA, and SNPs were used as instrumental variables (IVs) (23). The three principal hypotheses of a classical MR study were met for the overall process: exposure was directly influenced by IVs; IVs and confounders were not associated; through exposure, the risk of outcomes was directly influenced by IVs. Informed consent and ethical approval were available in the original studies. The present study was conducted in accordance with the latest STROBE-MR guidelines (24).

### Ethical approval

The MR study was conducted using GWAS summary statistics. All GWAS-based data are accessible to the public and freely downloadable on the GWAS Catalog (https://www.ebi.ac.uk). Ethical approval was obtained for each dataset.

### Ivs

All genetic variants that were related to RA (*P* < 5 × 10^−8^) were considered as IVs. SNPs in a linkage disequilibrium (LD) state were identified through testing of the corresponding LD. These independent SNPs were selected by removing those within a 10,000 kb window and a threshold *r*^2^ < 0.001. In order to exclude the potential pleiotropy effect, the secondary phenotype of each SNP was searched in the PhenoScanner V2 database, indicating whether SNPs would be potentially associated with confounders or risk factors for outcomes. SNPs that did not correspond to phenotypes related to outcomes were further analyzed, while the remaining SNPs were excluded. The variance *R*^2^ was calculated for the screened SNPs, and the strength of IVs was evaluated using F-statistics. This avoided weak-tool bias (23). Additionally, a meticulous calculation method (*F* = *R*^2^(N-K-1)/[K(1-*R*^2^)] was adopted, where *R*^2^ indicates cumulative explained variance of the selected SNPs during exposure, N refers to the number of samples for the selected GWAS, and K refers to the number of SNPs for the final analysis. If *F* > 10, the correlation between IVs and exposure was considered strongly enough, and the results of the MR analysis were not affected by the weak tool bias.

To ensure consistency of alleles of all SNPs between MI and RA, the SNP-RA and SNP-MI statistics were unified. Different outcomes, such as arrhythmia, AF, and IHD were adjusted. In the MR analysis, the primary analytical method used in accordance with heterogeneity was the IVW method as described previously (23). Simultaneously, to explore and deduce causality comprehensively, maximum-likelihood, MR-pleiotropy residual sum and outlier (MR-PRESSO), MR-Egger, median weighting, and MR-robust adjusted profile score (MR-RAPS) were also utilized (25). Each method made different assumptions related to the efficacy of IVs. When half of IVs were invalid, median weighting was estimated. MR-RAPS corrected horizontal multiplicity and reduced its deviation using robust adjusted contour scores. MR-Egger method had a low statistical ability, while it provided an estimation after correcting multiple effects. Outliers in IVW linear regression were automatically detected and removed by the MR-PRESO method for corrected MR estimation (25).

### Analysis of pleiotropy and heterogeneity

In order to perform sensitivity analysis, various methods were employed. Heterogeneity among individual SNP estimates was evaluated by the Cochran's *Q* test. It was proved as an appropriate method for analysis. If *P >* 0.05, the fixed-effects IVW method was considered as no heterogeneity was indicated; otherwise, the random-effects model was applied. In order to determine the horizontal pleiotropy of IVs, the MR-Egger method was also utilized (18). The average horizontal pleiotropic effect among SNPs was estimated by the MR-Egger method. The estimated IVW was considered to be accompanied by bias if *P*-value was less than 0.05. The probability of a SNP was also estimated by leave-one-out sensitivity test. Finally, the existence of pleiotropy was directly detected by drawing funnel and forest plots. A two-sided *P*-value less than 0.05 was considered statistically significant.

The statistical analysis was carried out using R 4.1.2 software, which included the “MR-PRESSO”, “Two-Sample MR”, and “MR.RAPS” packages.

## Results

### Causal relationship between the risk of AF or arrhythmia and genetic susceptibility to RA

As shown in Table 2, the prevalence of arrhythmia (odds ratio (OR) = 0.100, 95% confidence interval (CI): [0.100–1.000], *P *= 0.873) in the RA group was not significantly different from that in the control group (Supplementary Figure S1). The results were consistent with those obtained by the IVW method. Meanwhile, no significant association was found between the risk of RA and AF [OR = 1.003, 95% CI: (0.985–1.021), *P *= 0.755] (Supplementary Figure S2, Table 2).

**Table 2:** MR estimates of rheumatoid arthritis associated with the risk of cardiovascular diseases.

Disease	Methods	SNPs (*n*)	OR	95% CI	*P*-value
Arrhythmia	MR-Egger	58	1.0001	0.999554–1.000586	0.790956
	Weighted median	58	1.0003	0.999852–1.000668	0.211671
	IVW	58	0.9999	0.999650–1.000297	0.873395
	Simple mode	58	1.0002	0.999100–1.001278	0.735234
	Weighted mode	58	1.0001	0.999729–1.000475	0.593739
AF	MR-Egger	87	0.9963	0.968953–1.024419	0.794687
	Weighted median	87	1.0037	0.98574–1.022004	0.68795
	IVW	87	1.0028	0.985097–1.02091	0.755284
	Simple mode	87	0.9871	0.948271–1.02755	0.528316
	Weighted mode	87	1.0016	0.985722–1.017744	0.844586
MI	MR-Egger	82	1.0663	1.022345–1.1122	0.003724
	Weighted median	82	1.0680	1.025758–1.111929	0.001394
	IVW	82	1.0458	1.017061–1.075379	0.001636
	Simple mode	82	1.0480	0.947907–1.158437	0.363216
	Weighted mode	82	1.0685	1.031256–1.107087	0.000447
	MR-Egger	62	1.0010	1.00038806–1.001619716	0.00222287
	Weighted median	62	1.0006	0.999978267–1.001158133	0.059059183
IHD	IVW	62	1.0006	1.000244475−1.001040326	0.001551915
	Simple mode	62	1.0001	0.998491356–1.001828674	0.852802432
	Weighted mode	62	1.0006	1.000027694–1.001155086	0.044033966

### Causality of genetic susceptibility to RA on the risk of MI and IHD

As presented in Table 2, the results of the IVW method indicated that RA was correlated with the increased risk of MI and IHD. In addition, the prevalence of MI in RA cases was 1.046-fold that of the control group [95% CI: (1.017–1.075), OR = 1.046, *P *= 0.0016] (Supplementary Figure S3), and increase of the log-transformed OR of RA by one unit elevated the risk of IHD [95% CI: (1.000–1.001), OR = 1.004, *P *= 0.002] (Supplementary Figure S4). MR analysis of RA and MI indicated that the results of the weighted median analysis were highly consistent with those of the IVW method. The MR analysis of RA, MI, and IHD showed that the results of the MR-Egger analysis were highly consistent with those obtained by the IVW method.

### Analysis of horizontal pleiotropy and heterogeneity

As shown in Table 3, a series of methods were employed for MR analysis regarding the correlation of RA with arrhythmia, AF, MI, and IHD, in order to determine the presence of significant horizontal pleiotropy and heterogeneity in the present study. First, *P*-value was >0.05 in the heterogeneity test, demonstrating that SNPs had a negligible heterogeneity (Table 3). The fixed-effects IVW method was dominant in the MR analysis. The “leave-one-out” sensitivity analysis demonstrated that IVs involved in the present study had a negligible influence on the results (Supplementary Figure S5), and the funnel plot illustrates an asymmetric distribution of single IVs (Supplementary Figure S6), suggesting that the causality might not be affected by the potential bias.

**Table 3:** Testing of heterogeneity and pleiotropy of the RA-associated IVs from CVD GWAS-based data.

Outcomes	Pleiotropy test			Heterogeneity test					
	MR-egger			MR-egger			Inverse-variance weighted		
	Intercept	SE	*P*	Q	Q_−_df	Q_−_*P*-val	Q	Q_−_df	Q_−_*P*-val
Arrhythmia	−1.92 × 10^−05^	4.055 × 10^−05^	0.6385	76.14	56	0.0380	76.44	57	0.0438
AF	0.001	0.002	0.5484	221.27	85	4.3468 × 10^−05^	222.22	86	5.2687 × 10^−05^
MI	−0.004	0.004	0.2325	106.34	80	0.0261	108.27	81	0.0232
IHD	−7.63 × 10^−05^	5.061 × 10^−05^	0.1369	49.25	60	0.8376	51.53	61	0.8010

### Cardiovascular risk factors did not influence the development of RA

Overall, as cardiovascular risk factors may influence RA development and strengthen the conclusion, reverse MR analysis was further performed. As shown in Table 4, the prevalence of RA in the arrhythmia group was not significantly different from that in the control group. Meanwhile, no significant association was found between the risk of AF, MI, IHD, and RA risk (Supplementary Figure S7).

**Table 4:** Mr estimates of arrhythmia, AF, MI, and IHD on the risk for RA.

Disease	Method	SNPs (n)	*P*-value
Arrhythmia	IVW	5	0.3123275
	Weighted median	5	0.4035608
	MR-Egger	5	0.2961895
	Weighted mode	5	0.2066364
	Simple mode	5	0.5366099
AF	IVW	104	0.9380365
	Weighted median	104	0.6131080
	MR-Egger	104	0.6701567
	Weighted mode	104	0.5305349
	Simple mode	104	0.6706936
MI	IVW	12	0.485987
	Weighted median	12	0.075750
	MR-Egger	12	0.120265
	Weighted mode	12	0.272258
	Simple mode	12	0.139946
IHD	IVW	3	0.576534
	Weighted median	3	0.100359
	MR-Egger	3	0.129855
	Weighted mode	3	0.299454
	Simple mode	3	0.251141

## Discussion

The MR analysis was performed in the present study to explore the exact causality between RA and IHD based on large-scale GWAS. This study included multiple factors related to CVD, such as arrhythmia, AF, MI, and IHD. It was proved that RA increased the risk of CVD secondary to MI and IHD as opposed to AF and arrhythmia.

IHD in coexistence with RA is a proven main cardiovascular cause of death, which commonly imposes the highest disease burden among all CVDs worldwide (2). High BMI, diabetes mellitus (DM), hypertension, high cholesterol level, and smoking are some of the various risk factors involved (3). However, high systolic blood pressure, high LDL-C level, and smoking are the three main contributors to the mortality of IHD (1). Apart from traditional Framingham risk factors, gender (26, 27) and psychosocial factors (28, 29) are noteworthy. In 1997, Wallberg-Jonsson et al. found that overall mortality due to CVD and IHD were elevated in seropositive RA (30). Studies reported that RA was closely associated with a higher risk of IHD. RA patients were at a higher risk of IHD than those without RA (16.6% vs. 12.8%) (6). A meta-analysis concluded that there was a 48% increase in the risk of CVD in patients with RA (9). A high risk of CVD-related death (50%) was reported in patients with RA, and 59% in those with IHD (11). To date, no significant difference has been found in the traditional Framingham risk factors between patients with and without RA. After excluding traditional risk factors for CVD, Esther Houri Levi et al. demonstrated that RA was the main cause of CVD after RA (6). From a conventional aspect, inflammatory factors, such as von Willebrand factor (VWF), induced CVD through endothelial damage/dysfunction, platelet activation, hypercoagulability, and angiogenesis in patients with RA (31, 32). It was also speculated that the pathogenesis of CVD in RA patients was different from that in non-RA patients.

Previous studies identified RA as an independent risk factor for CVD. The causal relationship between RA and CVD has still remained elusive. For instance, patients with RA were not at the higher risk of hyperlipidemia than general population, which was the “lipid paradox” (33), while a higher risk of CV events was found in patients with RA compared with individuals without RA (33, 34). In recent studies, researchers used the MR analysis, and it was revealed that patients with RA were at the increased risk of coronary artery disease (CAD), angina, hypertension, heart attack, arrhythmia, and stroke (10, 35). Kessler et al. concluded that IHD, non-IHD, and arrhythmia were the leading causes of cardiovascular complications in RA patients (36). The results of the present study indicated RA did not increase the risk of arrhythmia and AF. Various risk factors identified for arrhythmia and AF were age, genetic predisposition, BMI, hypertension, DM, obstructive sleep apnea, HF, MI, and smoking. It was speculated that chronic systemic inflammation was the leading cause of arrhythmia in patients with RA (37). Some scholars also showed that severe acute respiratory syndrome coronavirus (SARS-CoV-2) increased the risk of arrhythmia (38). It was demonstrated that ischemia and the cardiac autonomic nervous system play a vital role in the modulation of cardiac arrhythmogenesis (39). It is easier to understand that cardiac autonomic nerve system dysfunction causes arrhythmia than to explore inflammatory etiologies. Inflammation was not reported as a major cause of arrhythmia in diabetic patients (40). In RA patients, vagus nerve stimulation inhibited cytokine production, which was a type of “inflammatory reflex” (41). Arrhythmia is only a protective reflex, while it may not merely cause CVD in RA patients. Thus, it remains unclear whether inflammation is a driver or a secondary factor for arrhythmia in patients with RA (42).

An increased risk of IM and IHD in RA patients is consistent with previously reported findings (21). Studies assessed the risk of CAD in RA patients (35). RA patients had a different IHD pathophysiology compared with non-RA patients (43). Coronary microcirculation dysfunction was more likely to cause insidious ischemia than atherosclerosis (44). Recent studies have shown that a genetic variant of the RARB gene (rs116199914) contributed to the development of subclinical atherosclerosis in patients with RA. Moreover, potential influences of the previously described cardiovascular risk loci NFKB1, MSRA, and ZC3HC1 were confirmed (45). Furthermore, other genetic polymorphisms in RA may increase the risk of CVD, including human leukocyte antigen (HLA) and related genes, tumor necrosis factor (TNF) superfamily genes, cytokines-related genes, chemokines-related genes, adipokines-related genes, and other genes (46). Neither anti-rheumatic drugs nor pro-inflammatory cytokine antagonists reduced the risk of CVD in RA patients (47). Therefore, inflammation and endothelial injury were not compelling enough to explain the risk of CVD in RA patients. Ischemia resulting from coronary microcirculation dysfunction may be the main cause of CVD. Exploring the mechanism of coronary microcirculation dysfunction in RA patients may be therefore advantages in preventing early cardiac dysfunction.

There were some limitations in the present study. Firstly, multiple methods were used to access the pleiotropy, although potential multiplicity might still be existed. Secondly, there might be a genetic diversity among different races. Thirdly, lower OR values were obtained compared with other studies. The clinical significance of the OR value remains to be further documented by the next studies.

## Conclusions

In conclusion, the present study suggested that there was an increased risk of IHD and MI in patients with RA. This MR study might provide new genetic evidence for the causal relationship between RA and the risk of CVD. The findings revealed that the control of RA activity could reduce the risk of CVD. Prevention of early cardiac dysfunction should be promoted through exploring the mechanism of coronary microcirculation dysfunction in RA patients.

## Data Availability

The original contributions presented in the study are included in the article/**Supplementary Material**, further inquiries can be directed to the corresponding author/s.

## Supplementary material

SUPPLEMENTARY FIGURE 1

Mendelian randomization analysis of RA and the risk of arrhythmia.

SUPPLEMENTARY FIGURE 2

Mendelian randomization analysis of RA and the risk of AF.

SUPPLEMENTARY FIGURE 3

Mendelian randomization analysis of RA and the risk of MI.

SUPPLEMENTARY FIGURE 4

Mendelian randomization analysis of RA and the risk of IHD.

SUPPLEMENTARY FIGURE 5

The MR “leave-one-out” sensitivity analysis of RA on arrhythmia, AF, MI, and IHD. (A) Arrhythmia. (B) AF. (C) MI. (D) IHD.

SUPPLEMENTARY FIGURE 6

Funnel plots of RA with arrhythmia, AF, and MI, IHD. The X-axis represents odds ratio (OR), and the Y-axis represents standard error (SE). (A) Arrhythmia. (B) AF. (C) MI. (D) IHD.

SUPPLEMENTARY FIGURE 7

MR testing of arrhythmia, AF, MI, and IHD associated with RA. The estimate of intercept can be interpreted as an estimate of the average pleiotropy of all SNPs, and the slope coefficient provides an estimate of the bias of the causal effect. (A) Arrhythmia. (B) AF. (C) MI. (D) IHD.

SUPPLEMENTARY TABLE 1

SNPs used to analyze the causal relationship between RA and arrhythmia, AF, MI, and IHD.
