# Supplementary material for: Relationship between rheumatoid arthritis and cardiovascular comorbidity, causation or co-occurrence: A Mendelian randomization study
Source: Front Cardiovasc Med. 2023 Mar 17;10:1099861. doi: 10.3389/fcvm.2023.1099861 (PMC10063906; doi:10.3389/fcvm.2023.1099861)
Supplement: Supplementary file 1 [file Datasheet1.pdf]

Beijing Etop Science & Technology Co., Ltd

EDITORIAL CERTIFICATE

This document certifies that the manuscript listed below was edited by proper English language, grammar, punctuation, spelling, and overall style by one or more of the highly qualified native English-speaking editors at Beijing Etop Science & Technology Co., Ltd.

Manuscript title:

Relationship between Rheumatoid Arthritis and Cardiac Comorbidity, causation or co-occurrence: A Mendelian Randomization Study

Authors:

Min Wang, Ce Chao, Kun Mei, Dongmei Di, Yongxiang Qian, Bin Wang, Xiaoying Zhang

Date Issued:

Beijing Etop Science & Technology Co., Ltd 1101051626465

February 13, 2023

This document certifies that the manuscript listed above was edited by proper English language, grammar, punctuation, spelling, and overall style by one or more of the highly qualified native English-speaking editors at Beijing Etop Science & Technology Co., Ltd. Neither the research content nor the authors' intentions were altered in any way during the editing process. Documents receiving this certification should be English-ready for publication; however, the author has the rights to accept or reject our suggestions and changes. If you have any questions or concerns about this edited document, please contact us at postmaster@etopsci.com.

Beijing Etop Science & Technology Co., Ltd. Provides a range of editing, translation and manuscript services for researchers and publishers throughout the world. Our top-quality PhD editors are native English-speakers from the top universities of the English-speaking countries. Our editors possess the highest qualifications to edit research papers written by non-English speakers. For more information about our corporation, please visit www.etopsci.com.
