# Supplementary figures and images for: Relationship between rheumatoid arthritis and cardiovascular comorbidity, causation or co-occurrence: A Mendelian randomization study
Source: Front Cardiovasc Med. 2023 Mar 17;10:1099861. doi: 10.3389/fcvm.2023.1099861 (PMC10063906; doi:10.3389/fcvm.2023.1099861)

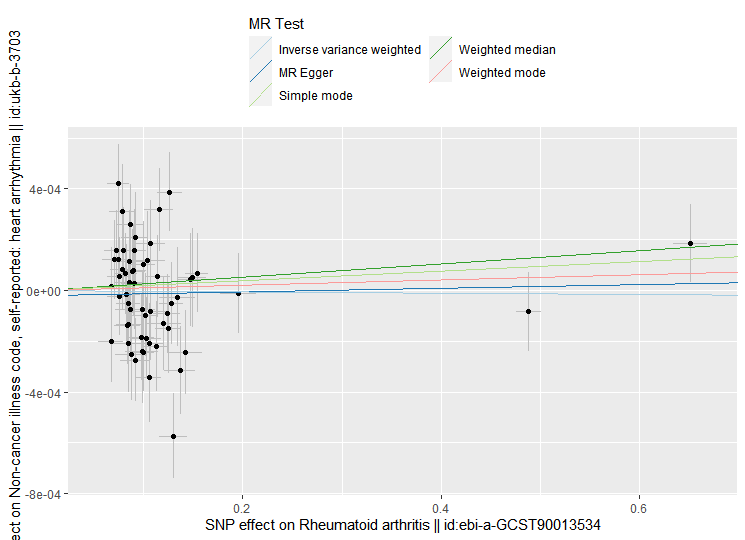

Supplement: Supplementary file 2 [file Image1.png]

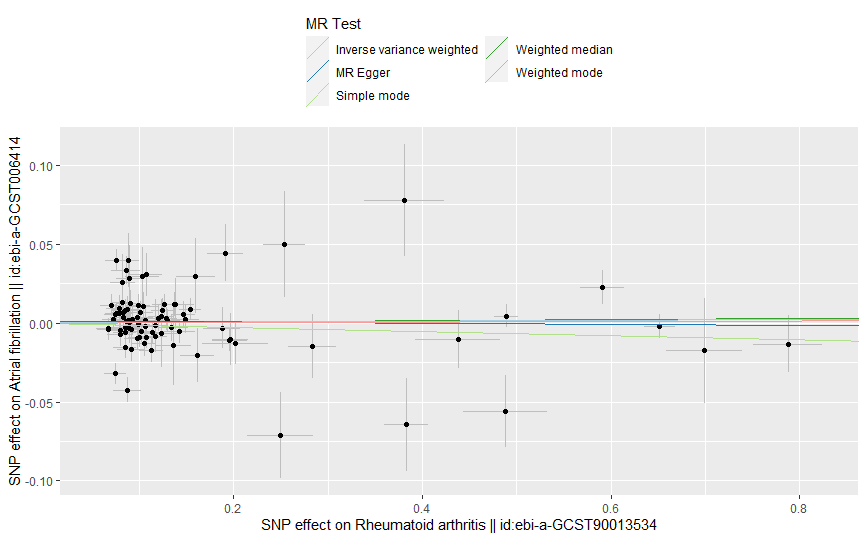

Supplement: Supplementary file 3 [file Image2.png]

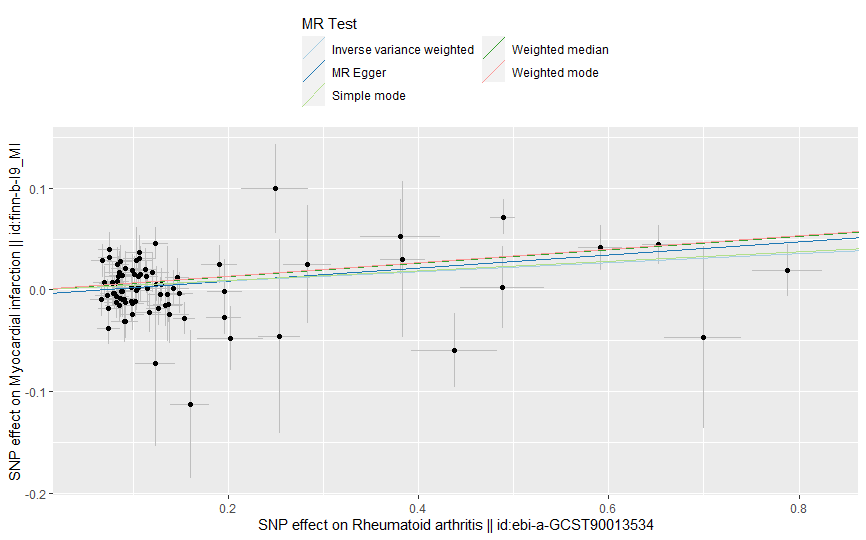

Supplement: Supplementary file 4 [file Image3.png]

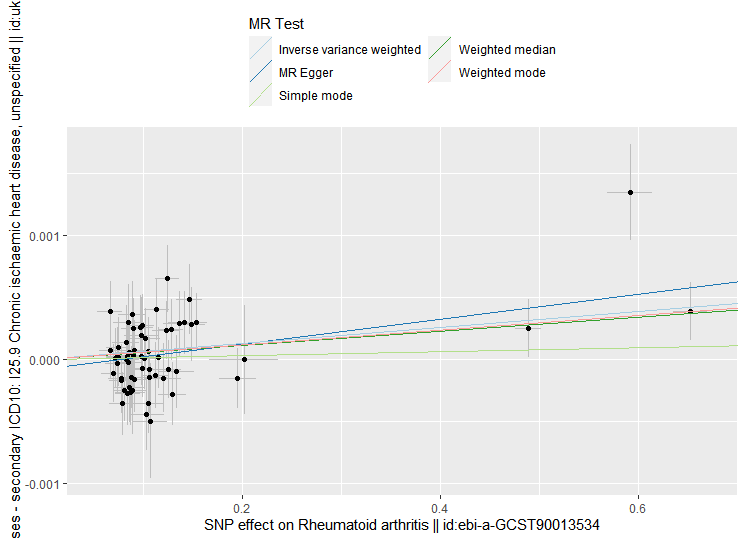

Supplement: Supplementary file 5 [file Image4.png]

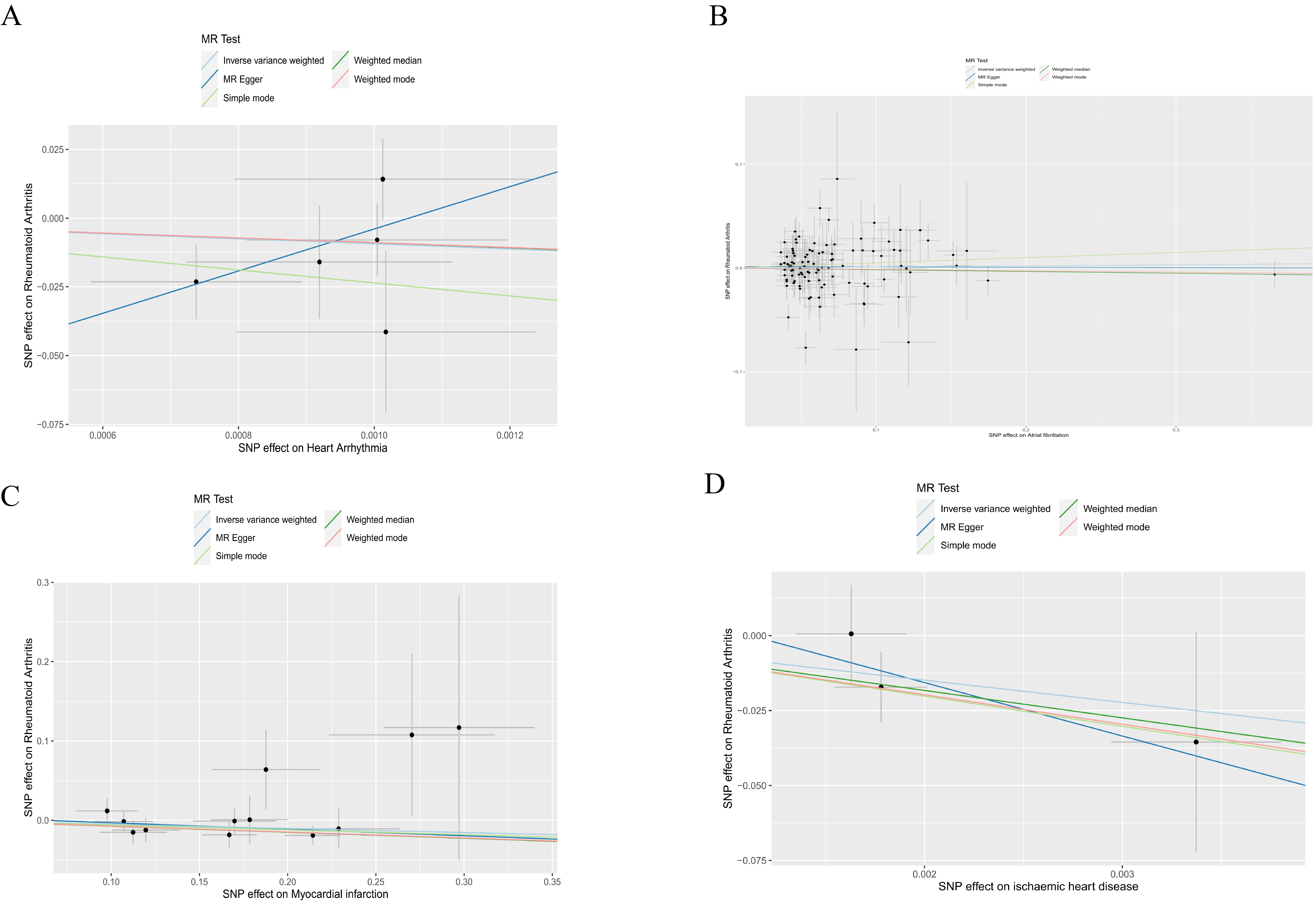

Supplement: Supplementary file 8 [file Image7.png]
